# Prevalence, mortality and healthcare economic burden of tuberous sclerosis in Hong Kong: a population-based retrospective cohort study (1995–2018)

**DOI:** 10.1186/s13023-020-01517-2

**Published:** 2020-09-25

**Authors:** William Ching-Yuen Chu, Lorraine Lok-Wing Chiang, Dorothy Chi-Ching Chan, Wilfred Hing-Sang Wong, Godfrey Chi-Fung Chan

**Affiliations:** Department of Paediatrics and Adolescent Medicine, The University of Hong Kong, Hong Kong, SAR, People’s Republic of China

**Keywords:** Tuberous sclerosis, Epidemiology, Prevalence, Mortality, Survival, Treatment, mTOR inhibitor, Healthcare burden, Healthcare resources, Hong Kong

## Abstract

**Background:**

We aim to elucidate the disease impact by accounting the prevalence, survival rate, genetics, mTOR inhibitor use and direct costs of tuberous sclerosis complex (TSC) in our local setting. TSC patients with documented visits to our local public hospitals in 1995–2018 were identified. The public hospitals captured most if not all local TSC patients. Demographics such as age, sex, death, genetic profiles were retrieved from the central electronic database. Data including prevalence, age distribution and survival rate were analysed. Direct cost was calculated with reference to the drug use and number of visits to various public hospital facilities.

**Results:**

We identified 284 surviving TSC patients (55.3% male) in Hong Kong. The age range was from 4.5 months to 89.9 years, with a median age of 27.2 years. Paediatrics (< 18 years) to adult (≥18 years) ratio was 1:2.84. The overall prevalence of TSC patients was 3.87 in 100,000 (i.e. 1 in 25,833). Genetically, TSC1:TSC2 ratio is 1:2.7.

Thirty seven patients died within the study period. The age of death ranged from 7.6 years to 77.8 years, with a median age of death at 36.6 years (IQR: 24.7–51.1 years). Most patients survived till adulthood. Survival rate at 20 and 50 years follow-up was 98.6 and 79.5% respectively.

Two hundred and twenty nine TSC patients (71.3%) had neurological manifestations, sixteen patients (5.0%) had chronic kidney diseases and five patients (1.6%) had pulmonary lymphangioleiomyomatosis. Forty seven (16.5%) TSC patients were prescribed with mTOR inhibitors within the study period. Healthcare facility utilization was further analysed in the 2008–2018 cohort. In particular, the mean number of specialist out-patient clinic visits per patient-year was 9.23 per patient-year, which was 4.91 times more than that of local general population.

**Conclusions:**

Prevalence of local TSC patients is within the range of that reported in the literature. Local TSC patients have fair long term survival, but they require disproportionally high healthcare cost when compared with the general population, particularly in terms of outpatient (OP) visits. Although effective disease-modifying agent (i.e. mTOR inhibitor) is available, it was not widely used yet in Hong Kong despite the fact that Government approved and supported its use recently. Further research on quality of life and setting up a comprehensive patient registry are necessary for more accurate assessment of cost and benefit.

## Background

Tuberous sclerosis complex (TSC) is a rare autosomal dominant genetic disorder resulting from mutation in one of the two TSC genes that encodes for the protein Hamartin on chromosome 9q34 (*TSC 1*) or Tuberin on chromosome 16q13 (*TSC2*) [1–3]. The loss of function gene mutation will affect the suppressive effect on genetic signal leading to the increase in the activation of the mammalian target of rapamycin (mTOR) pathway. The consequence is the uncontrolled cellular proliferation. This regulatory abnormality predisposes patients to tumour growth in multiple organ systems, such as brain, skin, retina, heart, kidneys and lungs [4–6]. Prior studies have demonstrated individuals with TSC2 mutation have overall more severe symptoms than those with TSC1 mutation [7, 8]. That includes more frequent and severe seizure, mental retardation (moderate and severe), autism, cortical tubers, renal angiomyolipoma, retinal hamartoma, and advanced facial angiofibroma [7, 9, 10]. These disease presentations would not only contribute to a considerable amount of healthcare cost, but might also bring about psychosocial burden to the family members and carers [11].

While TSC patients could develop multisystem life-threatening clinical manifestations [12], mTOR inhibitors (i.e. Sirolimus and Everolimus) are found to suppress the progression of multiple disease specific manifestations such as brain or kidney tumours [13], hence are widely used. Since 2018, Everolimus has been approved by the Food and Drug Administration and European Medicines Agency for treatment of refractory seizures in patients aged 2 years and older [14]. Everolimus has also been approved for treatment of patients aged ≥3 years with renal angiomyolipoma (AML) and TSC-related subependymal giant cell astrocytoma (SEGA) [15, 16]. While Sirolimus has been approved for treating lymphangioleiomyomatosis (LAM) in Japan and USA since 2014 and 2015 respectively [17], prior clinical studies also supported the use of Sirolimus for TSC patients with renal AML [18, 19].

Understanding the epidemiology and disease burden of TSC is crucial for better management of the TSC population [20]. Six population-based studies regarding the prevalence and mortality of TSC in the recent 15 years can be found (Table 1) [12, 21–26]. Amongst the six studies, three studies feature the prevalence of TSC in Caucasian population, with variable prevalence rate (ranges from 1 in 7872 to 1 in 24,956). Whereas for Asian population, population-based prevalence studies of TSC patients can only be found in Taiwan but it varies significantly with time (prevalence estimated 1:2,500,000 in 1997, 1:95,136 between 2004 and 2006, and 1:63,291 in 2010) [22, 24]. In terms of survival, only two studies from Mayo Clinic in 1991 and Bath in 2017 addressed the mortality issue [12, 27, 28]. In Bath’s report, the median age of death was 33 years (IQR: 26–46 years) with no cases died in the paediatric age range (< 16 years). In these surviving cohorts, there was no significant sex predisposition.

**Table 1:** Studies featuring the prevalence and mortality of TSC in the previous 15 years

Area (year of publication)	Prevalence (per 100,000)	Mortality	Case number
Northern Ireland (2006) [21]	1: 24,956 (4.00) in 2004	N/A	73 patients
Taiwan (2009) [22]	1: 95,136 (1.05) in 2006	N/A	208 patients
Quebec, Canada (2015) [23]	1: 7872 (12.7) in 2011	N/A	1004 patients
Taiwan (2016) [24]	1: 2,500,000 (0.04) in 1997 & 1: 63,291 (1.58) in 2010	N/A	471 patients
Sweden (2017) [25]	1:18,577 (5.38) in 2013	N/A	551 patients
Bath TSC Clinic, UK (2017) [12]	N/A	median age of death was 33 years (IQR 26–46)	284 patients

As a multisystem-involved genetic disease, the morbidity and treatment costs are significant predictors to burden on the health service providers. Parameters such as inpatient (IP) stay, Accident and Emergency department (A&E) and outpatient (OP) clinic visits are useful for assessment. However, the overall disease burden and financial implication remains undetermined especially under different health care financing systems [29, 30]. There is a knowledge gap existing between the epidemiology and healthcare utilization pattern [20, 30], which will affect the optimal management of TSC population on a population-based policy making level.

For Asia, there is inadequate data regarding the epidemiology and disease burden of TSC. Locally, despite effective target therapy being available, there is no comprehensive study on the demographics and health burden of TSC so far. Therefore, a population-based study is deemed necessary as a potential reference for both local and regional health care providers. Hopefully, this can serve as a benchmark for future healthcare planning and implementation.

## Methods

### Source of data

Data was retrieved from Clinical Data Analysis and Reporting System (CDARS). CDARS is an electronic database monitored by the local Hospital Authority, which is responsible for public healthcare services and captured almost all in- and out-patient information [31].

All patients were recorded in an unlisted and anonymous manner in the CDARS database by a unique reference key, which is known as CDARS ID number.

The total population and age-specific population data in Hong Kong were retrieved from Hong Kong by-census dated in the end of 2016 in calculating the prevalence rate.

Clinical records pertaining to the genetic diagnosis of TSC patients who were once followed up in the Department of Paediatrics and Adolescent Medicine of Queen Mary Hospital were also obtained. The Department of Paediatrics and Adolescent Medicine in Queen Mary Hospital takes care of 52 TSC patients and provides clinical genetic service in Hong Kong.

### Data recruitment

Cases of Tuberous Sclerosis Complex from 1 January 1995 to 24 September 2018 were identified from the CDARS using the 9th version of the International Classification of Disease and Related Health Problems (ICD-9) of 759.5. In addition, searching for potentially undiagnosed cases that meet the major and minor features specified in Updated Diagnostic Criteria for Tuberous Sclerosis Complex 2012 [32] was attempted. Retrieved variables include number of subjects, age, sex, ethnicity, mortality (as defined by death recorded in the Hong Kong Death Registry), genetic diagnosis (as from clinical records), mTOR inhibitor prescription, number of A&E attendance, OP attendance, operation (OT) records, lengths of stay in general ward and intensive care unit (ICU). Their clinical manifestations, including brain neoplasm (such as SEGA), mental retardation, developmental delay, pulmonary LAM, renal neoplasm requiring total or partial nephrectomy, chronic kidney disease (CKD) and the number of them on dialysis, were identified on CDARS using their corresponding ICD-9 codes.

### Statistical analysis

Current patient population is defined as surviving patients on the date of data retrieval (i.e. 24th September 2018), which, in other words, are the patients whose death dates were not recorded during the study period. Demographic information of the current patient population was analysed. This included number of patient population, male: female ratio, distribution by age and prevalence.

In the calculation of overall and age-specific number of cases, the patients were identified by their unique reference keys so as to eliminate double-counting of the same patient with multiple admissions into hospitals under the public hospital system. Overall and age-specific prevalence rate was calculated by the number of surviving TSC patients divided by the overall Hong Kong population obtained from 2016 by-census [33]. We also calculated the paediatrics-to-adult ratio, where paediatric patients were defined as patients under 18 years of age on the date of data retrieval while adult patients were defined as those aged 18 or above. For clinical manifestation, the prevalence of epilepsy was reflected by the number of patients who have been prescribed with anti-epileptic drugs over our study period.

Genetic diagnosis was obtained as a small-scale sampling. Demographic analysis regarding TSC1:TSC2 ratio was calculated and the number of those whose genetic results were negative was reviewed.

Cases of deceased TSC patients (i.e. patients with death dates recorded during the study period) were counted. The median and IQR of the overall and gender-specific death ages were recorded. The survival curve was expressed with Kaplan-Meier survival plot.

Patients who were prescribed with mTOR inhibitors were identified with mTOR inhibitors prescribed being counted according to their types. For direct cost analysis, TSC patients in the last 10 years of the study period (i.e. 25 September 2008 to 24 September 2018) were identified. The total hospital length of stay and ICU length of stay (both counted as inpatient-days) per patient-year were also calculated. These figures were compared with the corresponding figures of general population in the past year, as obtained in Hospital Authority Annual Report 2018–2019 [34]. After which total cost and cost per patient-year were estimated according to fees and charges of public hospital services provided by Hospital Authority starting from 18 June 2017.

Statistical analyses were done with SPSS version 24.0.0.0 (IBM SPSS Statistics).

## Results

### Patient characteristics and prevalence of TSC patients in Hong Kong

We identified 321 patients who were under the care of Hong Kong Hospital Authority hospitals, with 284 surviving patients. The overall prevalence of TSC patient is 3.87 in 100,000, using the Hong Kong Census data at the end of 2016 [33]. Among the surviving TSC patients, the male to female ratio is 1: 0.81 (male cases 157, female cases 127) (Table 2).

**Table 2:** Basic Demographics of TSC patients in Hong Kong

Study period	1-1-1995 to 24-9-2018
Total population^a^	7,336,585
Total number of TSC cases identified	321
Ethnicity, n (%)	
Chinese	299 (93.1%)
Non-Chinese	22 (6.9%)
Total number of surviving TSC patients ^b^	284
Prevalence (in 100,000)^b^	3.87
Male: female ratio^b^	1:0.81
Paediatrics (< 18 years): adult (≥ 18 years)^b^	1:2.84
Neurological, renal & lung manifestations, n (%)	
Epilepsy	229 (71.3%)
Mental retardation	101 (31.5%)
Developmental delay	46 (14.3%)
Brain neoplasm (including SEGA)	30 (9.3%)
CKD (overall)	16 (5.0%)
CKD (requiring dialysis)	4 (1.2%)
Renal neoplasm requiring nephrectomy	10 (3.1%)
Pulmonary LAM	5 (1.6%)
Genetic testing, *n* (%)^c^	
No mutation identified	3 (9.1%)
*TSC1* mutation	8 (24.2%)
*TSC2* mutation	22 (66.7%)
Both *TSC1* and *TSC2* mutations	0

^a^From 2016 by-census [33]

^b^On the date of data retrieval i.e. 24-9-2018

^c^Molecular testing for genetic mutations was based on the data of 33 patients

### Age distribution of TSC patients in Hong Kong

Their ages ranged from 4.5 months to 89.9 years, with a median age of 27.2 years. Paediatrics to adult ratio was 1:2.84. When dividing into age groups in 5-year ranges, the 20–24 age group had the largest total number of patients, accounting for 14.8% of the total surviving TSC patients. Prevalence among 15–19 age group (9.68 in 100,000) was the highest among all age groups. (Table 3).

**Table 3:** Distribution of TSC patients by age on 24-9-2018

Age group	Number of TSC patients (% of the total TSC population)	Population	Prevalence (X in 100,000)
0–4	12 (4.2%)	279,470	4.29
5–9	26 (9.2%)	291,767	8.91
10–14	19 (6.7%)	259,218	7.33
15–19	33 (11.6%)	340,907	9.68
20–24	42 (14.8%)	445,074	9.44
25–29	35 (12.3%)	510,236	6.84
30–34	30 (10.6%)	577,232	5.20
35–39	19 (6.7%)	571,293	3.33
40–44	14 (4.9%)	569,805	2.46
45–49	17 (6.0%)	567,037	3.00
50–54	13 (4.6%)	643,065	2.02
55–59	5 (1.7%)	623,049	0.80
60–64	12 (4.2%)	495,279	2.42
65–69	4 (1.4%)	395,703	1.01
70–74	2 (0.7%)	220,827	0.91
75–79	0 (0%)	206,374	0
80–84	0 (0%)	166,928	0
85 or above	1 (0.4%)	173,281	0.57
**Total**	**284 (100%)**	**7,336,585**	**3.87**

### Number of TSC patients with neurological, renal and pulmonary manifestations

For neurological manifestations, 229 (71.3%) TSC patients had epilepsy, 101 (31.5%) patients had intellectual disability, 46 (14.3%) patients had developmental delay, and 30 (9.3%) TSC patients had brain tumour (e.g. SEGA). For renal manifestations, 10 (3.1%) patients had renal neoplasm requiring partial or total nephrectomy. A total of 16 (5.0%) patients were documented with CKD, among which 4 (1.2%) patients were dialysis-dependent. For pulmonary manifestations, 5 (1.6%) patients had LAM, and they were all females.

### Genetic diagnosis of TSC patients in Hong Kong

Fifty-two patients who had a clinical diagnosis of TSC and followed up in Queen Mary Hospital, Hong Kong were identified. Molecular testing for genetic mutations was available in 33 patients. Of these, 22 patients (66.7%) had a TSC2 gene mutation, 8 (24.2%) had a TSC1 gene mutation, none had both TSC1 and TSC2 gene mutations and 3 (9.1%) had no mutation identified.

### Death rate and survival of TSC patients in Hong Kong

**Fig. 1:**
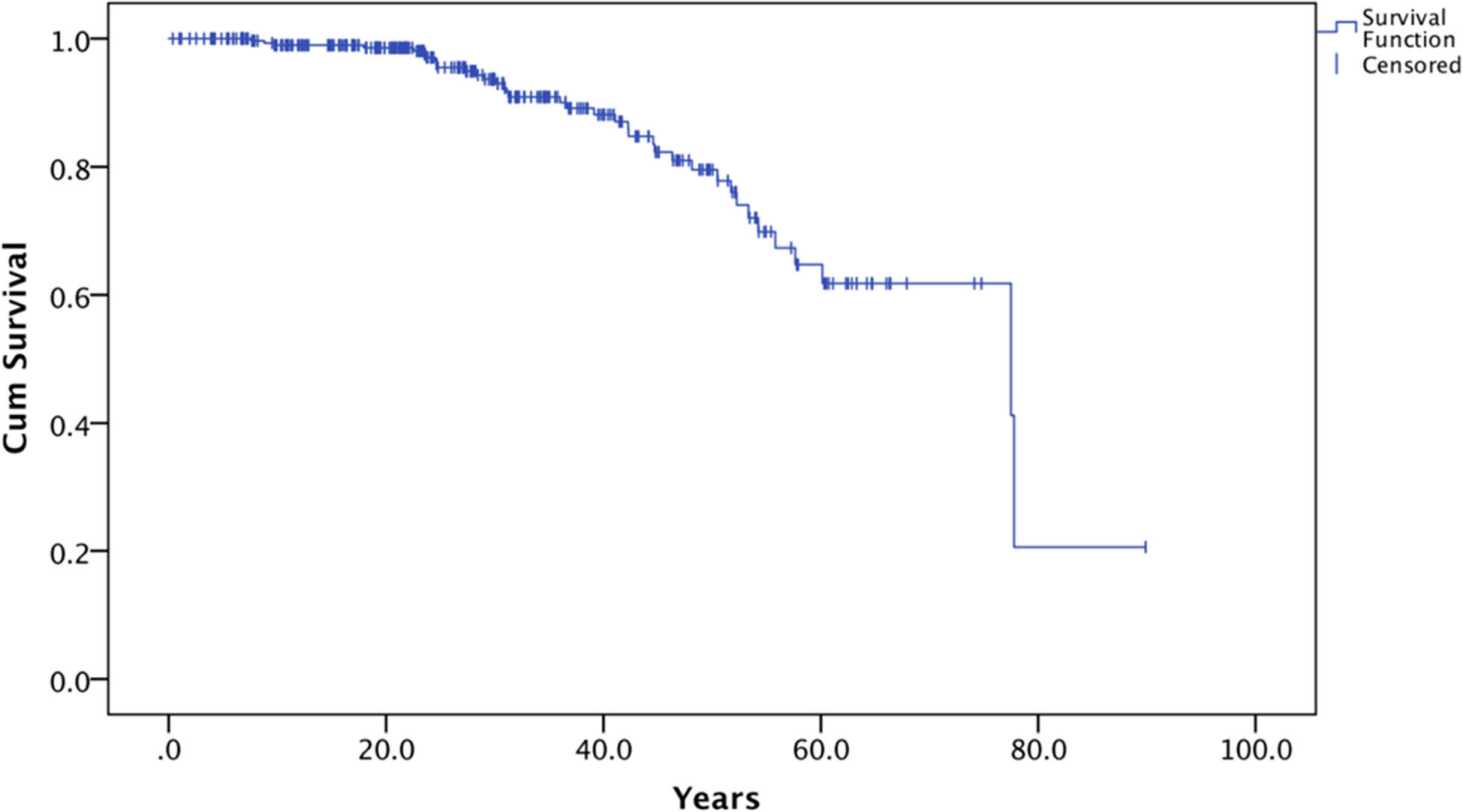
Kaplan-Meier survival plot for overall survival of local TSC patients

Thirty-seven patients died within the study period. The age of death ranged from 7.6 years to 77.8 years, with a median death age of 36.6 years (IQR: 24.7–51.1 years). Most patients survived till adulthood. The survival rate at 20 years old and 50 years old were 98.6 and 79.5% respectively (Fig. 1), and the mean survival time was 67.4 years (95% CI: 61.6–73.2). This is shorter than the expected age of > 83 yrs. for local normal population. There was no significant difference in survival time and mean age of death between male and female.

### mTOR inhibitor prescription in TSC patients

Forty-seven (16.5%) TSC patients were prescribed with mTOR inhibitors within the study period, of which 16 had Everolimus only, 20 had Sirolimus only, 11 switched between Everolimus and Sirolimus

### Healthcare utilization analysis in 2008–2018 cohort

Further analysis was performed using 25th September 2008 – 24th September 2018 cohort, which included 304 TSC patients. A majority of the patients required A&E (244 patients, 80.3%), OP visits (289 patients, 95.1%) and hospital admission (227 patients, 74.7%) within this period, and 41 (13.5%) and 100 (32.9%) of them required ICU admission and operation respectively. Among those who underwent operation, 12 of them received operation related to kidney neoplasm or for dialysis, and 10 of them received operation for brain tumour excision or obstructive hydrocephalus management. The mean hospital length of stay per patient-year was 9.36 days, which is higher than that of the general population (5.9 days). Hospital admission of TSC patients accounted for HKD45,227.2 (USD5,819) per patient-year. A noticeable length of stay in hospital were attributed by ICU stay, as the mean ICU length of stay per patient-year was 0.112 days, which required HKD2,737.0 (USD352) per patient-year. There were 0.734 A&E visits, 9.23 OP visits per patient-year, which were 2.48 times and 4.91 times of that of the general population respectively (0.296 and 1.88 visits respectively). A&E and OP visits required HKD903.1 (USD116) and HKD10,989.1 (USD1,414) per patient-year respectively. The total and mean cost per patient-year on these domains are summarized in Table 4. Of note, these values accounted for less than 10% of the actual expenses because the Government subsidized more than 90% of the public hospital cost.

**Table 4:** Summary in healthcare resources utilization of TSC patients

	A&E	ICU (days)	OP	OT	IP (days)
Total visits in 10 years	2232	341	28,073	190	28,466
Service usage PPPY (no. of times compared with general population [34])	0.734 (2.48 times)	0.112	9.23 (4.91 times)	0.063	9.36 (1.59 times)
*Service charge* *[HKD]*	***$1230 per attendance*** ***(USD158)***	***$24,400*** ***per day*** ***(USD3,139)***	***$1190 per attendance*** ***(USD153)***		***$4830*** ***per day*** ***(USD621)***
Total cost in 10 years [HKD]	$2,745,360 (USD353,200)	$8,320,400 (USD1,030,450)	$33,406,870 (USD4,297,910)		$137,490,780 (USD17,688,656)
Cost PPPY [HKD]	$903.1 (USD116)	$2737.0 (USD352)	$10,989.1 (USD1,414)		$45,227.2 (USD5,819)

*A&E* Accident and Emergency department, *ICU* Intensive care unit, *OP* Out-patient clinic, *OT* Operation, *IP* In-patient admission, *PPPY* Per patient per year

## Discussion

Data on the population-based epidemiology and healthcare burden of ethnic Chinese or Asian TSC patient are lacking. This study is a territory-based study describing the epidemiology of TSC patients in Hong Kong over a period of 23.7 years (from 1995 to 2018).

The local disease burden of TSC patients is reflected by the disease prevalence, survival profile and direct costs. The utilization of CDARS allows complete capture of all TSC cases under the care of our public health system, which handles over 80% of all admission in Hong Kong. In particular, most patients with chronic illness are followed up by the public health system. Moreover, CDARS is an electronic system of all medical records under the Hong Kong Hospital Authority [31]. Previous local studies have demonstrated accurate ICD-9 coding and completeness of territory-based data retrieved from this system [35, 36]. The population-based nature of this study in the calculation of prevalence and healthcare utilization minimized the selection bias which could occur in studies conducted in a specific referral centre.

Our study presents a number of novel findings which fill the knowledge gap on the prevalence and survival profile of local TSC patients. In particular, the age-specific survival rate of TSC patients is highlighted. Survival profile of our local TSC patients was favourable, with nearly all of them survived beyond 20 years old. The median mortality age and the IQR of mortality age were corroborated by the 2017 study in Bath TSC Clinic, United Kingdom (median mortality age: 33 years, IQR: 26–46 years) [12]. Their study reported no death before the age of 16. This difference could be attributed to the availability of a better management and care for TSC patients provided by a specialized TSC clinic in the UK, which is not available in Hong Kong. However, both studies suggested that TSC patients could potentially enjoy a long life expectancy.

Another important parameter is the availability of mTOR inhibitors. Our data showed that it was not widely used in our TSC patients (47 patients, 16.5%). mTOR inhibitors (i.e. Sirolimus and Everolimus) are found to be effective in suppressing the overall disease progression of TSC, through inhibiting the mTOR pathway and anti-tumour effect [13]. The Hong Kong government has recently approved Everolimus for TSC treatment. However, there are certain prescription restrictions. The criteria for subsidized prescription are: [1] presence of astrocytoma or SEGA, [2] renal angiomyolipoma greater than 3 cm. Access to Everolimus by other TSC patients were restricted, limiting the choices to the cheaper mTOR inhibitor, Sirolimus alone. The mTOR inhibitor prescription, together with prevalence data shown in this study, would be informative to our government in terms of financial estimation of the cost required for mTOR inhibitors subsidies for TSC patients.

This study further delineates the direct cost attributed to TSC patients in the public hospital system, over a study period of 10 years. The results reveal a high healthcare burden disproportionate to its prevalence. Although disproportionately high inpatient cost compared to prevalence have been discussed in rare disease population in general [37, 38], there are several considerations in healthcare planning worth highlighting in the context of TSC. First, TSC patients have higher mean inpatient length of stay (9.36 days) when compared with the whole rare disease population in Hong Kong (6.1 days) [29]. Second, TSC patients have favourable survival, and adult TSC patients were nearly three times that of paediatric. Hence, that means that healthcare resources were required in both paediatric units and specialities in adult medicine units. Third, after comprehensively reviewing the healthcare service domains other than inpatient cost, an astonishing number of 9.23 OP visits per patient-year, being 4.91 times more than the number of general population, was noted. The number of A&E attendance was also noticeably higher than the general population. Consequently, these suggest that on top of the considerable inpatient requirement, A&E and OP clinic are particularly resource-intensive service domains in the management of TSC. This is compatible with our knowledge that TSC patients develop a number of life-threatening complications which require frequent hospital admissions and regular follow-ups [30, 39]. Our study also described the prevalence of local TSC patients with manifestations in the neurological, renal and pulmonary systems, which were each found to incur high burden of illness in a recent review [26]. With regards to the multisystem involvement, locally speaking, TSC patients were referred to multiple subspecialties separately when they exceed paediatric age. According to overseas experience, Auvin et al. [40] provided a 3-step guide in TSC multi-disciplinary team care, Bar et al. discussed the need for optimizing transition programs for TSC patients from paediatrics to adult care [41]. Hence, measures taken to centralize the OP service to TSC patients, such as training doctors in a multi-disciplinary team may optimize the healthcare resources provided to TSC patients.

We are aware of two other Taiwan studies in the published literature that estimated the epidemiology on a population level with the use of electronic healthcare system instead of cross-sectional respondents [29]. Comparing other epidemiology and direct costs studies of TSC, the prevalence found in our study (3.87 in 100,000) is higher than Taiwan’s studies (1.05 and 1.58 in 100,000 respectively) [22, 24]. The accuracies of their studies, however, could be limited by the shorter study periods (3 years and 13 years respectively), or cases that are outside the national health insurance database. With a long study period (23.75 years), we are confident our study depicts a less biased prevalent rate, suggesting that our results are of relevance to clinical and policy decision makers.

Comparing service utilization, we noted that the inpatient admission of our TSC patients (9.36 day per patient-year) is higher than overseas population-based studies (3.25 days in Sweden [25], 6.2 days in UK [39]). OP visit in Hong Kong (9.23 visits per patient per year) was also higher than that reported in Sweden (4.7) but lower than reported in UK (13.7). In Kingswood et al’s study, higher outpatient visit, GP visit, inpatient admission were noted on TSC patients in comparison with an age-, sex- and last record date-matched Comparator Cohort. Our study did not use a matched comparator cohort, because we aimed at finding out the actual service utilization and cost of TSC patients incurred in the healthcare system over 10 years, taken into account their age and sex. On the other hand, we took one more step forward in delineating A&E utilization of TSC patients compared with the general population. The results revealed that TSC patients required more A&E visits than the general population, which provided another prospective in viewing the disproportionate economic burden TSC patients posed on a healthcare system. We are aware that Rentz et al. and Reaven et al. [29] also reported high physician visits and A&E visits. Nevertheless, Rentz et al. was carried through recruiting questionnaire respondents who had internet access and received alert on a few patient advocacy websites; hence, selection bias towards TSC patients with higher cognitive function and less severe forms of the phenotype was highly probable. Besides, their study focused more on the comparison of direct cost in a claim-based manner. As with our study, we delineated the healthcare utilization attributed to TSC patients with the use of an electronic system capturing all medical records under the care of our public health system, over a study period of 10 years. Consequently, our data captured is likely to be of relevance to clinical and policy decision makers.

There are 4 broad caveats to the study findings. First, despite the use of an electronic system which captures all medical records under the care of our public health system, there might be a minimal chance of missing out TSC patients who had never attended local public hospitals. However, TSC patients often require referral to multiple specialists due to its multisystem manifestation, thus are less likely handled in private hospitals alone. In order to reduce the likelihood of missing cases, in the study design, we adopted a long retrospective study period (23.7 years). Therefore, we are confident that the potential uncaptured cases are minimal and did not systemically bias our results. Second, under-diagnosis of TSC clinically could be a major source of error that renders the prevalence in this study underestimated. Since the diagnosis of TSC in local centres was mainly by clinical findings, failure of diagnosis might be possible. Given the broad spectrum of disease manifestation and severity, the identification of TSC in mildly affected individuals could be challenging [42]. To further improve the accuracy of prevalence estimation, we also expanded the search criteria. A search using the combination of the diagnostic codes of the major and minor features listed in Updated Diagnostic Criteria for Tuberous Sclerosis Complex 2012 [32] was performed; however, there was no additional cases identified from the CDARS system. With no previously undiagnosed cases could be found even with the expansion of search criteria, there are unlikely to be systematic biases in under-diagnosis of TSC locally. Third, the disease burden of individual manifestations of TSC might not be reflected by CDARS accurately. As the causes of death of the deceased were not documented in CDARS, our study could not identify potential life threats for TSC patients according to age. We attempted to capture the prevalence of major neurological, renal and pulmonary manifestations, which were well-reported to cause significant burden of illness. However, it is possible that mild manifestations were not documented by clinicians into CDARS. Thus, the prevalence of individual clinical manifestations of TSC reported in this study is prone to underestimation. Prevalence of epilepsy is reflected by the number of patients documented with anti-epileptic drug use, so it is also subjected to underestimation. Fourth, data on genetic diagnosis was only obtained from one hospital, as not all patients had genetic diagnosis available. Whether such information is representative for all local patients requires further verification.

## Conclusions

The prevalence and genetic testing of TSC1 and TSC2 mutation of local TSC patients are comparable to the literature. The prevalence data serves as an important reference for healthcare financing, such as estimating the cost of the prescription of Everolimus, which was enlisted as a special drug under the local Hospital Authority Drug Formulary in 2019 [43]. Subgroup analysis between paediatrics and adult population further provides information for healthcare cost calculation and planning in Hong Kong.

Local TSC patients have fair survival. However, they require disproportionally high mean healthcare cost per patient-year when compared with the general population locally and overseas TSC populations, particularly in terms of OP visits. Potential measures taken to centralize resources for TSC patients can be explored to optimize healthcare service planning. Also, despite effective target therapy being available, it was not widely used in Hong Kong. Further research on quality of life and a comprehensive patient registry are necessary for cost and benefit analysis. Such a registry will be important for a more thorough understanding of the natural history of TSC and facilitating centralization of healthcare resources for TSC patients.

## Data Availability

The datasets used and/or analysed during the current study are available from the corresponding author on reasonable request and with permission of the Hong Kong Hospital Authority.

## Abbreviations

TSC: Tuberous sclerosis complex
mTOR: Mammalian target of rapamycin
AML: Angiomyolipoma
SEGA: Subependymal giant cell astrocytoma
LAM: Lymphangioleiomyomatosis
ICD-9: The 9th version of the International Classification of Disease and Related Health Problems
CDARS: Clinical Data Analysis and Reporting System
IQR: Interquartile range
CI: Confidence interval
A&E: Accident and emergency
IP: Inpatient
OP: Outpatient
OT: Operation
ICU: Intensive care unit
CKD: Chronic kidney disease
PPPY: Per patient per year
